# Heating Differentiates Pecan Allergen Stability: Car i 4 Is More Heat Labile Than Car i 1 and Car i 2

**DOI:** 10.1002/fsn3.4747

**Published:** 2025-02-16

**Authors:** C. Nacaya Brown, Rebecca A. Dupre, Christopher C. Ebmeier, Shaina Patil, Brennan Smith, Christopher P. Mattison

**Affiliations:** ^1^ Food Processing Sensory Quality USDA Agricultural Research Service New Orleans Louisiana USA; ^2^ Oak Ridge Institute for Science and Education U.S. Department of Energy Oak Ridge Tennessee USA; ^3^ Department of Biochemistry University of Colorado Boulder Boulder Colorado USA

**Keywords:** allergen, allergy, antibody, heating, modification, pecan, protein, proteomic, walnut

## Abstract

Pecans are a staple in American cuisine and may be eaten raw but are often roasted or baked. Heating can alter pecan protein content and pecan allergen solubility. Three seed storage proteins (Car i 1, Car i 2, and Car i 4) commonly act as allergens and are recognized by IgE from pecan allergic individuals. Time resolved changes in the solubility of pecan allergens in response to heat were assessed by SDS‐PAGE, immunoblot, and mass‐spectrometry. Whole pecans from three different commercial sources were roasted for up to 24 min in an oven at 300◦F. Relatively smaller proteins such as Car i 1 remained soluble even after 24 min of heating and were stably observed by SDS‐PAGE, immunoblot, and mass‐spectrometry. However, the solubility of higher molecular mass proteins such as Car i 2 and Car i 4 decreased after 20 and 24 min of heating as reflected in SDS‐PAGE and decreased antibody binding on immunoblot. Nonetheless, mass‐spectrometric peptide characterization indicated that Car i 2 peptides remained relatively stable throughout heating. In contrast, Car i 4 was relatively more sensitive to heating and produced relatively fewer heating‐insensitive peptides. A set of heat‐resistant peptides for the reliable detection of three pecan allergens, Car i 1, Car i 2, and Car i 4, were identified.

## Graphical abstract

Oven heating differentiates pecan allergen protein and peptide stability and solubility. The Car i 1 protein and Car i 2 peptides are more heat stable compared to Car i 4. Car i 2 heating‐induced fragments remain soluble, while Car i 4 is relatively more sensitive to heating‐induced insolubility.

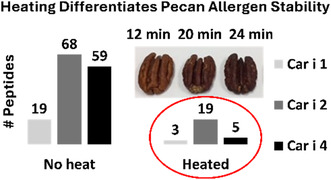

## 1 Introduction

Pecans ( *Carya illinoinensis* ) are native to North America and are an important part of U.S. agricultural production. Several pecan varieties are grown across a wide swath of the Southern United States. According to the USDA, pecan consumption in the U.S. met its highest value in 2019 with a record 65,842 tons or the equivalent of 0.2 g per person. U.S. pecan nut production was valued at ~$650 million and ~ $600 million in 2022 and 2023, respectively (NASS 2023, 2024). Additionally, increases in pecan production were also recorded in 2019 and for the 2020–2021 marketing season at 255 million and 302 million pounds, respectively. Projected pecan consumption and production rates for 2023–2032 are estimated to grow from 2.4 billion to 3.5 billion (IMARC 2023). Consumer demand informs production rates, and the versatility and diversity of products produced from pecan trees, nuts, and husks makes the pecan industry a lucrative business.

Pecans and other tree nuts are regarded as components of a healthy diet, but they are also among a group of eight foods that commonly cause serious allergic reactions (Suther et al. 2020). Food allergies can result in severe life‐threatening reactions and cause adverse economic and social effects on families (Ruchi et al. 2018). Food allergies are mediated by immunoglobulin E (IgE) antibody binding to specific allergenic proteins in foods. There are three seed storage proteins in pecans (Car i 1, Car i 2, and Car i 4) that commonly act as allergens. Car i 1 is a small (approximately 13 kDa) protein in the albumin family composed of two subunits held together by a conserved network of four cysteine disulfide bonds (Sharma et al. 2011a). Car i 2 and Car i 4 are members of the cupin superfamily. Car i 2 is a 55 kDa trimeric vicilin (Zhang et al. 2016), and Car i 4 (55.4 kDa) is a hexameric legumin (Sharma et al. 2011b).

Pecans and walnuts are part of the *Juglandaceae* family in the *Fagales* order, and pecans are native to North America. Pecan and walnut allergens are homologous and often cross‐react (Goetz, Whisman, and Goetz 2005; Elizur et al. 2020; Smeekens, Bagley, and Kulis 2018; Brettig et al. 2021). A European study demonstrated that only 75% of walnut allergic children had coexistent pecan allergies, but 97% of children with pecan allergy were also sensitive to walnut (Brough et al. 2020). However, the study cited geographical, population size, and preferred consumption‐based differences in sensitization among the children tested. Linear peptide assays and homology‐based approaches have been used to predict and characterize the immunoreactive determinants and IgE epitopes within pecan and walnut allergens (Sharma et al. 2011a, 2011b; Minkiewicz, Mattison, and Darewicz 2022).

Heating can alter food allergen potency by affecting protein solubility, altering protein structure, or inducing chemical modifications on proteins (Masthoff et al. 2013). For example, heating‐induced changes in peanut and tree nut solubility have been characterized (Verhoeckx et al. 2015). Heating has been shown to alter the IgE‐reactivity of peanut allergens Ara h 1 and Ara h 2 (Vissers, Blanc, et al. 2011). Further, heating‐induced modifications on arginine and lysine residues can alter the ability of digestive and endolysosomal enzymes to cleave peanut allergens (Mattison et al. 2015). Heating‐induced modification of the immunodominant cashew nut allergen Ana o 3 has also been documented, but the immunological significance of the modification is not known (Mattison, Grimm, et al. 2017).

Characterization of heated pecan samples with rabbit polyclonal anti‐pecan sera indicated that pecan allergens were generally stable, but that longer heating times resulted in decreased antibody binding (Mahesh Venkatachalam et al. 2006). However, historical reports indicate that storing or heating pecans may lead to increased allergen potency or content and may potentially create neo‐allergens that can lead to allergic reactions in some individuals that are not observed with fresh pecan samples (Berrens 1996; Malanin and Lundberg 1995). In the current study, heated pecans were carefully evaluated for changes in protein solubility and antibody binding, and trypsin‐treated extracts were assessed by mass‐spectrometry for differences in peptide content and amino acid modification.

## 2 Experimental Methods

### 2.1 Materials and Reagents

Different brands of pecan nuts were purchased from three local grocery stores. Sequencing grade trypsin for LCMS‐MS analysis was obtained from Promega (Madison, WI, USA). Invitrogen 10%–20% tricine gels, 4X NuPAGE LDS sample buffer, and Safe Stain were obtained from Life Technologies (Carlsbad, CA, USA) and used according to the manufacturer's recommendations. Precision Plus Protein Dual Color molecular weight standards were purchased from Bio‐Rad (Hercules, CA, USA). The rabbit polyclonal anti‐pecan antibodies were generated by Genscript (Piscataway, NJ, USA) using a borate pecan extract from store bought pecans without additional heating to immunize rabbits, as described in Clermont et al. (2023) (Clermont et al. 2023). Human pecan allergic volunteer sera were purchased from Plasma Lab International (Everett, WA, USA), and included two female and three male donors with pecan specific IgE CAP values of 7.13, 13, 1.61, 23.7, and 33.5 kU/L, respectively. Infrared dye‐labeled secondary antibodies and streptavidin were purchased from LI‐COR (Lincoln, NE, USA).

### 2.2 Extraction of Heated Pecan Samples

Shelled pecans were heated in groups of 10–12 nuts in a single layer on aluminum foil boats at 300°F (149°C) for 0 (control), 12, 20, or 24 min and then were allowed to cool to room temperature as described in Mattison et al. (2016) (Mattison et al. 2016). Pecan extracts were prepared from each sample of 10–12 unheated or heated nuts (approximately 13–16 g) by grinding the nuts in a food grade blender, followed by defatting with petroleum ether (1:4 m/v) twice at 50°C for two hours. The defatted protein samples were dried in a fume hood, and were then re‐ground and resuspended in a borate buffered saline solution (100 mM H_3_BO_3_, 25 mM NaB_4_O_2_, and 75 mM NaCl at pH 8.6) demonstrated to optimally solubilize nut proteins (Sathe et al. 2009) and stirred for 1 h at room temperature. The ratio of ground pecan to buffer was 1:10. Extracts were centrifuged for 15 min at 4°C and 16,873 rcf to remove suspended solids and collect the supernatant. Protein concentrations were determined by absorbance at 280 nm relative to BSA standards using a NanoDrop (ThermoFisher, Pittsburgh, PA, USA), and they ranged from 0.16–1.5 mg/mL. Protein signals from the 12 samples were compared by imaging of Coomassie‐stained SDS‐PAGE gel using an Odyssey CLx scanner and Image Studio software (LI‐COR, Lincoln, NE, USA) as described in Clermont et al. (2023). Soluble protein samples were stored at −80°C prior to use.

### 2.3 SDS‐PAGE and Immunoblot

Soluble protein extracts (2 μg) from the unheated and heated pecan samples were visualized via electrophoresis and subsequent immunoblotting. An appropriate ratio of water, loading buffer, and sample were mixed in the absence of dithiothreitol for each individual sample. Samples were vortexed and heated at 50°C for 5 min and centrifuged at high speed for 5 min. A 10 μL aliquot of each extract solution was added to each lane and electrophoresed at 125 mV for 90 min. Protein bands were transferred to a PVDF membrane. Following transfer, blots were blocked for 1 h at room temperature with phosphate buffered saline containing 0.1% Tween‐20 (PBST). Membranes were rinsed three times for 5 min each with PBST followed by the addition of a 1:1000 solution of rabbit anti‐pecan polyclonal antibodies. After a 30‐min dark incubation at room temperature, membranes were washed as above and a 1:10,000 solution of IRDye 680‐labeled donkey anti‐rabbit secondary antibody was applied and incubated for 30 min in the dark. Human IgE binding was detected with sequential addition of biotinylated anti‐human IgE (1:1000) followed by IRDye 680‐labeled streptavidin (1:10,000) with washing as described above in between. Immunoblot images were captured using a Licor Odyssey CLx (LI‐COR, Lincoln, NE, USA).

### 2.4 Liquid Chromatography‐Mass Spectrometry (LC–MS) Orbitrap Mass‐Spectrometry

#### 2.4.1 Pecan Extract Sample Preparation for Mass Spectrometry

Pecan extract samples were diluted in 50 mM Tris–HCl, pH 8.5 with 5% (w/v) SDS, reduced with 10 mM tris (2‐carboxyethylphosphine) (TCEP), alkylated with 40 mM chloroacetamide, and denatured by boiling at 95°C for 10 min. Each sample was digested using the SP3 method (Hughes et al. 2014). Carboxylate‐functionalized ‘speed beads’ (GE Life Sciences, Marlborough, MA, USA) were added to the lysates followed by 80% (v/v) acetonitrile. Bead‐bound proteins were washed twice with 80% (v/v) ethanol and twice with 100% acetonitrile. Lys‐C/Trypsin (Promega) was used to digest bead‐bound proteins overnight with gentle rotation at 37°C. Tubes containing the ‘speed beads’ were centrifuged, and then they were placed on a magnetic rack to facilitate removal of digested peptides in solution. Soluble peptides were desalted with an Oasis HLB cartridge (Waters) according to the manufacturer's instructions and dried under vacuum.

### 2.5 Mass Spectrometry Analysis

Samples containing 1 μg of tryptic peptides (measured with a NanoDrop spectrophotometer at A280 nm) resuspended in 3% (v/v) acetonitrile/0.1% (v/v) trifluoroacetic acid were injected onto a C18 1.7 μm, 130 Å, 75 μm X 250 mm M‐class column (Waters), using a Thermo Ultimate 3000 RSLCnano UPLC. A 2%–20% acetonitrile gradient at 300 nL/min over 100 min eluted peptides into a Q‐Exactive HF‐X mass spectrometer (Thermo Scientific). Precursor mass spectra (MS1) for each sample were captured with a resolution of 120,000 (spanning 380 to 1580 m/z) using an automatic gain control (AGC) target of 3E6 and a maximum injection time of 45 milliseconds. The isolation width for precursor peptide ions during MS2 fragment scans was set to 1.4 m/z, and the 12 most intense ions were sequenced. All MS2 spectra were captured at a resolution of 15,000 and higher energy collision dissociation (HCD) at 30% of normalized collision energy, with a target of 1E5 for AGC and a maximum injection time of 100 milliseconds.

Data were processed and searched using Mascot Distiller, with peptide and fragment mass tolerances set at 20 and 50 ppm, respectively. The digestion enzyme was specified as trypsin and up to two missed cleavages were allowed. A custom pecan allergen library containing translated Sumner and Pawnee transcriptome sequences was used to search for the proteins of interest (Clermont et al. 2023; Huang et al. 2017; Mattison, Rai, et al. 2017). In all searches, cysteine carbamidomethylation (delta m/z 57.021464) was included as a fixed modification and methionine oxidation (delta m/z 15.994915) as a variable modification. Error tolerant searches, performed in three iterations, were done to identify potential lysine and/or arginine modifications using the variable modifications listed in Table S1. Mascot Distiller's label‐free quantitation was used to align and integrate peptide peak areas. Unique/common peptides were determined using filtered true/false (from Mascot LFQ output) columns in Excel (a complete list of observed allergen peptides can be found in Table S2), and each allergen was analyzed independently (data for all Car i 4 isoforms was combined). The number of theoretical tryptic peptides from each pecan allergen was determined using https://web.expasy.org/peptide_mass/.

Heating‐induced changes in peptide occurrence were correlated to the average intensity of Car i 1, Car i 2, or Car i 4 peptides. For a given time point, the sum of individual protein intensities for Car i 1, Car i 2, and Car i 4 were separately calculated for each replicate, then averaged using Formula (1) as described in Carillo et al. (2010).

(1) $${\left(\sum \text{peptide intensities time}\;X,\mathrm{rep}\;1+\sum \text{peptide intensities time}\;X,\mathrm{rep}\;2\right)}^{*}\;0.5$$

For each protein at 12, 20, and 24 min of heating, the summed peptide intensity ratios (relative to 0 min heating) were plotted to compare abundance changes in each allergen with heating time.

The following criteria were applied to Car i 1, Car i 2, and Car i 4 to identify candidate marker peptides. First, peptides had to be detected at all time points (0, 12, 20 and 24). Second, they had to be observed at an intensity of ≥ 10^5^ at 24 min of heating. Third, they did not decrease by greater than two orders of magnitude in intensity during heating. Fourth, they did not contain missed cleavage sites (arginine or lysine). Selected peptides that met these criteria are displayed in Table 1.

**TABLE 1:** Potential pecan nut Car i 1, Car i 2, and Car i 4 marker peptides detected in all (unheated and heated) samples. The average peptide abundance for two biological replicates is displayed for each peptide at each time point.

**Candidate Pecan Marker Peptides**					
Car i 1					
	0	12	20	24	Epitope Reference
103‐QQQQEEGIR‐111	2.22E+05	2.78E+05	9.09E+05	8.42E+05	Sharma et al. 2011a
Car i 2					
47‐WEFQQCQER‐55*	1.63E+08	2.60E+08	2.14E+08	2.34E+08	NA
87‐EEIVDPR‐93*	1.43E+07	1.98E+07	3.11E+07	5.60E+07	NA
272‐YEQCQQQCER‐281	8.20E+06	1.09E+07	9.60E+06	6.71E+06	Minkiewicz, Mattison, and Darewicz 2022
285‐GQEQQLCR‐292*	2.82E+06	3.67E+06	4.56E+06	3.16E+06	Minkiewicz, Mattison, and Darewicz 2022
435‐DAESV**I**VVTR‐444*	1.62E+08	1.95E+08	2.69E+07	2.53E+07	Minkiewicz, Mattison, and Darewicz 2022
577‐SSGGPISLK‐585	6.97E+07	1.14E+08	3.20E+07	3.46E+07	Minkiewicz, Mattison, and Darewicz 2022
608‐QLQEMDVLVNYAEIK‐622	2.39E+08	2.07E+08	2.43E+06	3.41E+05	Minkiewicz, Mattison, and Darewicz 2022
624‐GAMMVPHYNSK‐634	2.09E+05	3.98E+05	8.56E+04	1.99E+04	Minkiewicz, Mattison, and Darewicz 2022
635‐[ATV]VVYVVEGTGR‐647	1.31E+08	1.76E+08	3.85E+06	3.17E+06	Minkiewicz, Mattison, and Darewicz 2022
751‐[EEI]EEIFER‐759*	2.92E+08	3.23E+08	1.02E+08	7.27E+07	Minkiewicz, Mattison, and Darewicz 2022
743‐ELSFNMPR‐750	3.19E+07	5.20E+07	2.49E+07	2.13E+07	Minkiewicz, Mattison, and Darewicz 2022
492‐LLQPVNNPGQFR‐503	3.45E+08	3.80E+08	3.94E+07	3.35E+07	Minkiewicz, Mattison, and Darewicz 2022
447‐ATLTFVSQER‐456*	2.10E+08	3.31E+08	1.01E+08	8.53E+07	Minkiewicz, Mattison, and Darewicz 2022
519‐VFSNDILVAALNTPR‐533	1.19E+09	1.40E+09	8.77E+07	4.44E+07	Minkiewicz, Mattison, and Darewicz 2022
Car i 4					
35‐FGECQLK‐41*	2.70E+07	2.10E+07	4.47E+06	7.26E+06	Sharma et al. 2011b
93‐FYLAGNPR‐100*	5.42E+07	6.94E+07	9.50E+06	2.00E+07	Minkiewicz, Mattison, and Darewicz 2022
404‐EGQVLTVPQNFAVIK‐418*	5.52E+08	3.80E+08	4.68E+07	4.20E+07	Sharma et al. 2011b
419‐EGQLLIIPQNFGVVK‐433*	6.20E+07	5.26E+07	3.34E+06	9.50E+05	Sharma et al. 2011b
447‐TNEDAMISPLVGR‐459*	2.08E+08	1.26E+08	3.41E+06	2.52E+06	Minkiewicz, Mattison, and Darewicz 2022
465‐AIPEEVLANA[FQIPR]‐479*	6.73E+08	3.87E+08	1.10E+07	8.91E+06	Minkiewicz, Mattison, and Darewicz 2022

*Note:* The epitope reference column indicates sequences matching, similar, or adjacent to linear or in silico predicted linear IgE epitopes.

Abbreviation: NA, no IgE epitope match.

* Indicates that the sequence is unique to pecan.

Car i 1, Car i 2, and Car i 4 peptides with heating‐induced arginine or lysine modifications were filtered to reveal those with an intensity of ≥ 10^4^, and these are shown in Table 2. To determine the percentage of arginine and lysine residues modified following heating, a calculation using Equation (2) was performed. The solution to Equation (2) shows the percentage of altered protein content attributed to heating‐induced arginine and lysine modifications. It is important to note that we are only considering the total detected pecan allergen peptides and not unassigned sequences.

(2) $$\begin{array}{c} \;\frac{\#R,K\;\text{Modified Peptides}}{\#\text{Total Detected Modified Peptides}\;\mathrm{at}\;\text{Selected Time}\left(s\right)}\\ \;\times 100=\%R,K\;\text{Modification} \end{array}$$

**TABLE 2:** Heating‐induced modifications observed within Car i 1, Car i 2, and Car i 4 peptides. The average peptide abundance for two biological replicates is displayed for each peptide at each time point. Bracketing “[]” indicates sequences matching, similar, or adjacent to linear or in silico predicted linear IgE epitopes.

Heating‐Induced Peptide Modifications					
Peptide	Modification	12	20	24	Epitope Reference
**Car i 1**					
103 – [QQQQEEGIrGEE MEEMVQCASDLPK]—127	(111) Hex (R)	1.90E+06	1.31E+06	1.17E+06	Sharma et al. 2011a
103 – [QQQQEEGIRGEEMEEM VQCASDLPkECGISSR]—134	(115) Oxidation (M); (118) Oxidation (M); (127) Hex (K)	2.40E+08	4.21E+06	3.15E+06	Sharma et al. 2011a
**Car i 2**					
378—S[SGGPISLkS]QR—389	(386) HydroxymethylOP (K)	2.00E+06	3.71E+06	2.12E+06	Minkiewicz, Mattison, and Darewicz 2022
740 – [EAkELSFNMPR]—750	(742) HydroxymethylOP (K); (748) Oxidation (M)	2.67E+07	2.81E+06	2.49E+06	Minkiewicz, Mattison, and Darewicz 2022
487—LEMVkLLQPVNN[PGQFR]—503	(489) Oxidation (M); (491) Carbamyl (K)	8.11E+05	3.65E+05	4.39E+05	Minkiewicz, Mattison, and Darewicz 2022
608—QL[QEMDVLVNYAEIKr]—623	(612) Oxidation (M); (623) Hex (R)	9.80E+05	3.82E+04	3.37E+05	Minkiewicz, Mattison, and Darewicz 2022
**Car i 4**					
404—EGQVLTV[PQNFAV]Ikk—419	(418) Carboxyethyl (K); (419) Carboxyethyl (K)	2.03E+05	1.03E+05	1.63E+05	Sharma et al. 2011b
419—kAGNQGFEWVAFK—431	(419) HydroxymethylOP (K)	1.52E+05	5.94E+04	6.86E+04	Sharma et al. 2011b

### 2.6 In Silico Protein Modeling

A model of Car i 1 was generated using Molecular Operating Environment software (MOE, 2020.0901, Chemical Computing Group, Montreal, QC, Canada) using the Car i 1.0101 (Car i 1.0101, GenBank Protein AAO32314 and UniProt Q84XA9) sequence. Ber e 1 (pdb 2LVF) residues 8–101 served as a template molecule for Car i 1 modeling (EHMMER score 6.2e‐21). Protonate3D and QuickPrep were used to prepare the structure, fill in missing atoms and small loops, cap termini, fashion disulfide bonds, permit terminal amides, sulfonamide, and imidazole groups to flip, insert hydrogens, and resolve ionization state to optimize hydrogen bonding.

## 3 Results

### 3.1 Heating Reduces Solubility of Higher Molecular Mass Pecan Proteins

**FIGURE 1:**
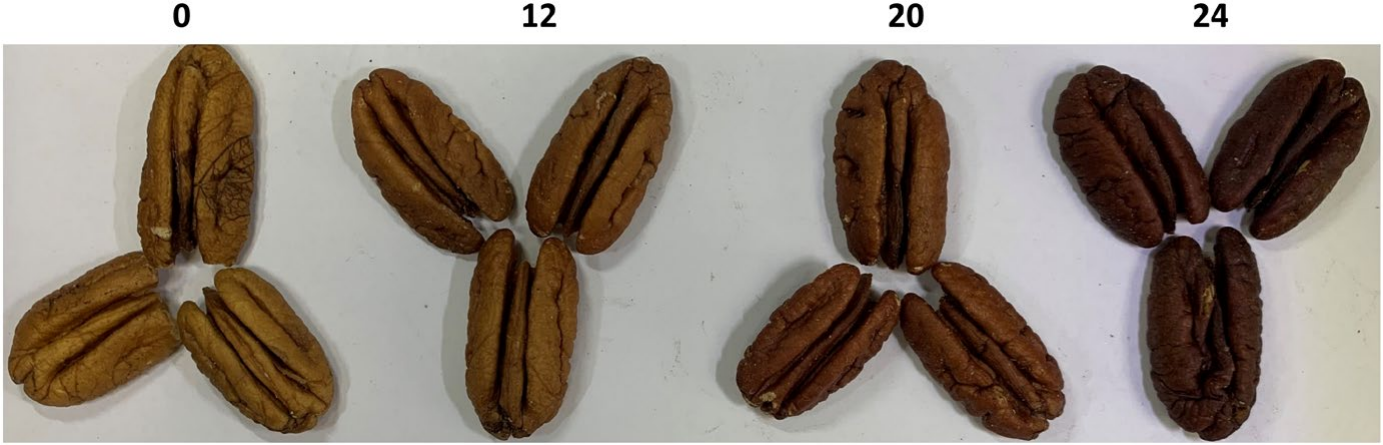
Representative image of oven roasted pecans. Pecans from three independent sources were oven roasted in a single layer on a parchment‐lined baking sheet for either 0 (raw control), 12, 20, or 24 min at 300°F.

Shelled pecans purchased from three different commercial vendors were oven roasted at 300°F for 12, 20 or 24 min. A non‐roasted control sample (‘0’) was reserved and termed “raw” in all treatments. The dark‐brown color of the heated pecan samples correlated with roasting time (Figure 1).

**FIGURE 2:**
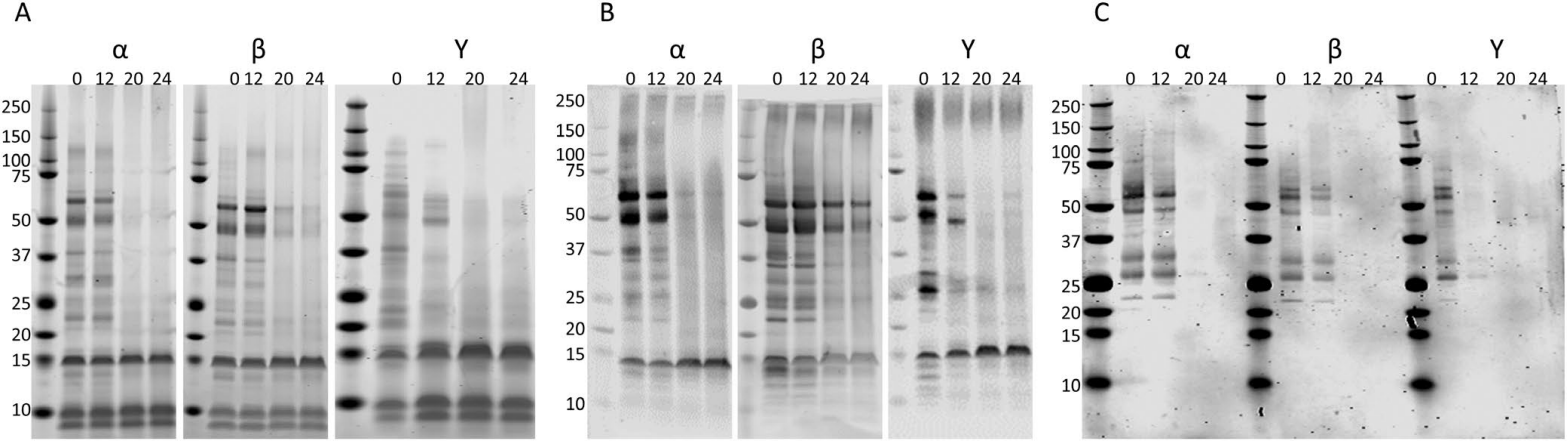
Characterization of heating‐induced changes in pecan allergen solubility. Changes in allergen content are reflected in SDS‐PAGE (A), rabbit anti‐pecan immunoblot (B), and human IgE anti‐pecan immunoblot (C) of pecan samples from three different commercial sources (α, β, and Υ) heated for 0, 12, 20, and 24 min (from left to right in each panel) at 300°F. Car i 1, Car i 2, and Car i 4 predicted masses are 16, 55, and 55.4 kDa (respectively), and molecular mass markers are indicated on the left of each panel.

The soluble extracts from the heated pecans were evaluated via SDS‐PAGE. Imaging of electrophoresed protein samples (5 μg per lane) revealed that the solubility of larger proteins decreased with time in each of the three samples (Figure 2A).

Immunoblotting with rabbit anti‐pecan antibodies revealed similar differences in the 0 to 24‐min time points with relatively larger proteins (> 25 kDa) decreasing in signal after 20 and 24 min of heating (Figure 2B). In contrast, the level of smaller protein bands (< 20 kDa) was similar among the treatments and varied only slightly with heating. When samples were probed with a pool of five human pecan allergic volunteer sera, there was also a heat‐dependent decrease in IgE pecan allergen binding (Figure 2C). This can be observed in the loss of recognition of bands migrating near the 50 kDa markers that likely correspond to the Car i 2 and Car i 4 allergens.

**FIGURE 3:**
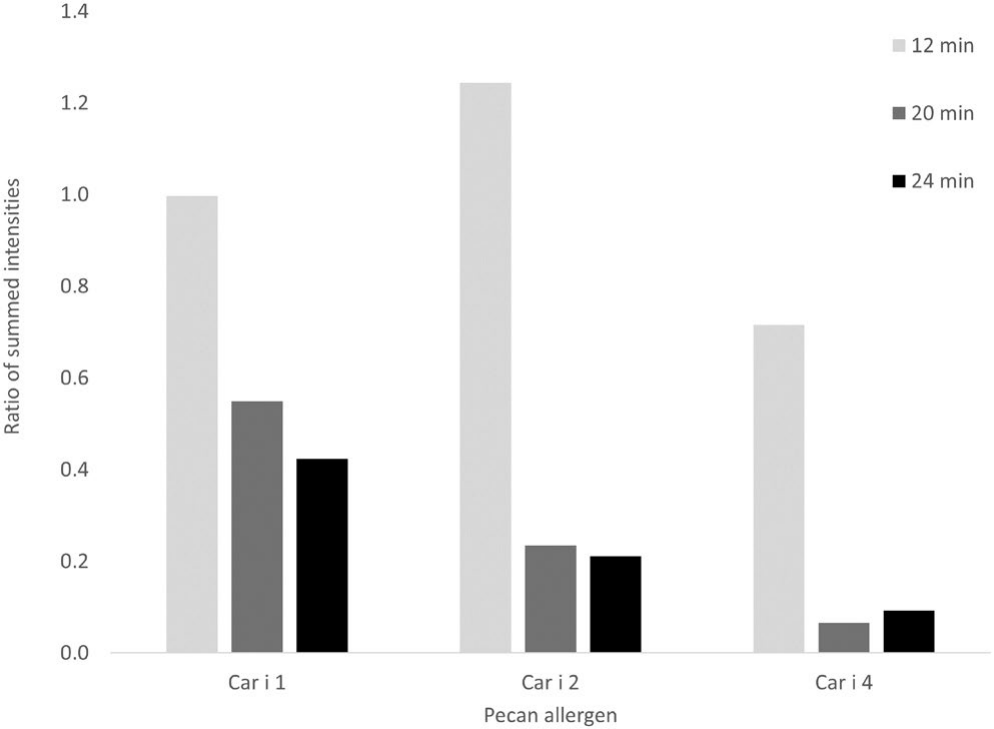
Heating‐induced changes in peptide intensity observed by Orbitrap mass‐spectrometry. The analysis was conducted using the total allergen intensities observed at 0, 12, 20, and 24 min. Ratios were calculated relative to the summed intensities of unheated samples.

Due to the similar trend of heating‐induced changes among the different commercial pecan samples, one was chosen for a more detailed analysis using mass‐spectrometry. For each time point, mass‐spectrometry was used to further characterize the changes in allergen solubility and/or stability following pecan heating via peptide observation (a complete list of observed allergen peptides can be found in Table S2). Following Orbitrap‐MS analysis, intensity‐based analysis was used to compare the 0–24 min heat‐induced differences in pecan proteins relative to the number of possible tryptic peptides for Car i 1, Car i 2, and Car i 4. Both modified and unmodified peptides were included in the analysis and summed intensity ratios at the respective timepoints were plotted. A correlation between heating time and relative protein abundance (based upon peptide observation) of each allergen was observed, but the data strongly suggested that Car i 1 peptides exhibited a more gradual decrease in peptide intensity at 20 and 24 min of heating compared to Car i 2 and 4 (Figure 3).

Additionally, Car i 4 peptide abundance changed more quickly than that of Car i 2 between 0, 12, and 20 min of heating. The decrease in the occurrence of measurable peptides in Car i 4 and Car i 2 relative to Car i 1 is consistent with the heating‐induced solubility decrease observed via SDS and immunoblot for proteins greater than 20 kDa (Figure 2). Two isoforms of Car i 4 were observed in the sample, and they produced essentially the same variation in overall peptide profile. Figure 2 data are plotted using only the Car i 4.0101 isoform for simplicity, and all further evaluations of Car i 4 protein content were performed using the Car i 4.0101 isoform.

### 3.2 Heating Reduces the Number of Stable Peptides

**FIGURE 4:**
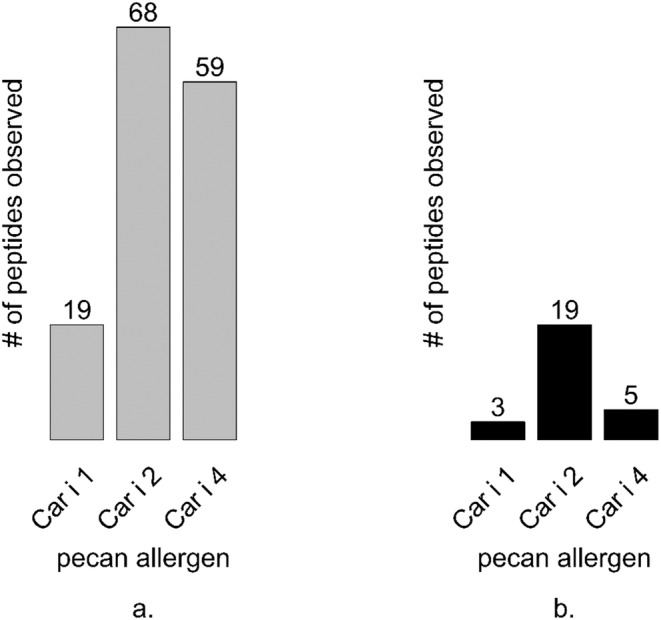
Stable peptide observation among untreated and heated pecan samples. The number of stable Car i 1, Car i 2, and Car i 4 peptides identified via Mascot search of Orbitrap data are shown for peptides occurring at all time points (A) and for peptides (B) occurring only in heated samples.

The effects of oven roasting on the protein content of pecan were further investigated to understand the distribution of the number of re‐occurring peptides detected between 0 and 24 min of heating. Overall, the total number of peptides identified was inversely correlated with increased heating. For example, an 84%, 72% and 94% reduction in peptide number was observed for Car i 1, Car i 2, and Car i 4, respectively (Figure 4). The analysis revealed the heat stable peptides for Car i 1, Car i 2, and Car i 4 that could potentially serve as robust pecan allergen peptide markers (Table 1).

Across all heating points, 146 potential marker peptides were identified, and 21 of them are shown in Table 1. Of these, 12 peptide sequences are unique to pecan only and are not shared with walnut or other tree nut allergens. Out of the 12 unique peptides, six sequences originated from Car i 4, six were attributed to Car i 2, whereas unique marker peptides not shared by walnut allergens were absent for Car i 1 (Table 1). In addition to the identification of marker peptides, we investigated the occurrence of peptides with heating‐induced modification in relation to heating‐induced insolubility, precipitation, or fragmentation.

### 3.3 Heating‐Induced Protein Modification Alters Protein Stability

One of the ways that heating can modify proteins is through direct chemical alteration of amino acid residues. Due to their hydrophilic nature as polar amino acids, arginine and lysine are often exposed on the surface of proteins, so mass‐spectrometry was used to screen for several types of arginine and lysine modifications within pecan allergens. Including the heated time points (12, 20, 24, and) the most abundant heating‐induced arginine or lysine modifications observed in order of decreasing occurrence include delta:H(2)C(3)O (1), hex, hydroxymethylOP, and carboxyethyl modifications. Respectively, these specific modifications represent 19%, 10%, 10%, and 8% (calculated via Equation (2)) of the total heating‐induced modifications detected.

**FIGURE 5:**
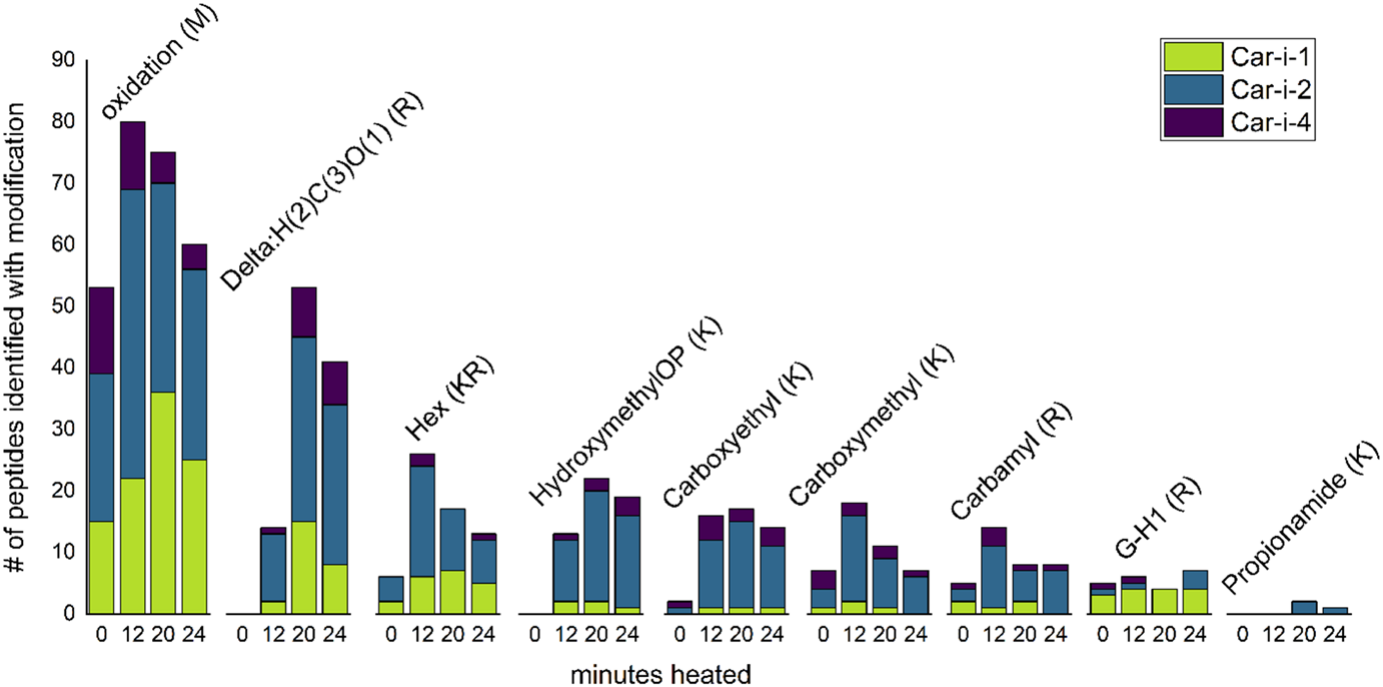
Heating‐induced peptide modifications identified by mass‐spectrometry. The number of (M) methionine oxidations, hex (K/R), hydroxymethylOP (K), carboxymethyl (K), delta:H(2)C(3)O (1) (R), carboxyethyl (K), carbamyl (K), G‐H1 (R), G‐H1 (R), and propionamide (K) modifications occurring at either 0, 12, 20, or 24 min, were calculated for each allergen. Each column represents the number of peptides (y‐axis) containing a specific modification at a given time point (x‐axis) observed in Car i 1 (green), Car i 2 (aqua), or Car i 4 (purple).

It should be noted that: (1) a peptide containing two delta:H(2)C(3)O (1) modifications is indistinguishable from a single hydroxymethylOP modification by mass spectrometry, (2) propionamide modification was inconsistently detected, and (3) G‐H1 modifications were consistently observed between 0 and 24 min for Car i 1 only (Figure 5). Heating‐induced modifications increased as the total number of stable peptides decreased. Although observation of individual modifications varied across stable peptides, an increase in the observance of methionine oxidation for Car i 1 and Car i 2 between 0 and 12 min of heating was followed by a decrease in the observance of methionine oxidation between 20 and 24 min (Figure 5). In contrast, decreased methionine oxidation of Car i 4 was observed beginning at 12 min and remained relatively constant between 20 and 24 min.

As summarized in Table 2, heating‐induced modifications were observed in Car i 1, Car i 2, and Car i 4. Irrespective of protein size, Car i 1 sustained the greatest percentage of R/K modified peptides relative to the total number of arginine or lysine residues present in each allergen (47% compared to 14% and 19% for Car i 2 and Car i 4, respectively). The R111 and K127 modifications within Car i 1 were consistently observed between 12 and 24 min of heating. The observed “QQQQEEGIrGEEMEEMVQCASDLPkECGISSR” peptide contained two missed cleavages (at R111 and K127) and was modified at K127:Hex. This fragment harbors two linear peptides (“QEEGIRGEEMEE” and “GIRGEEMEEMVQ”) that have been reported to strongly react with pecan allergic IgE (Sharma et al. 2011a). Heating‐induced modifications within Car i 2 and Car i 4 were also observed scattered throughout the length of the proteins (Table 2). The Car i 2 peptide, “QLQEMDVLVNYAEIKR”, contains a 16 amino acid long sequence that overlaps with an IgE reactive sequence (“QEMDVLVNYAEIKRGAMMVPHYNSKATVVVYVVEGTGRYEMACP”) in the orthologous Jug r 2 homolog. The Car i 4 peptide, “EGQVLTVPQNFAVIKK”, is a predicted epitope based upon similarity to the walnut Jug r 4 allergen (Sharma et al. 2011a, 2011b) (Minkiewicz, Mattison, and Darewicz 2022). Further, the Car i 4 fragment, “PQNFAV”, overlaps with an in silico predicted Jug r 4 epitope, “LTIPQNFAVVKRARNEGFEWVSF”, and results in 26% coverage of the complete walnut epitope.

## 4 Discussion

Previous reports have demonstrated both the stability of pecan allergens following heating, and the potential for generation of heating‐induced neo‐allergens. Soluble heated pecan extracts from different commercial sources were evaluated using several molecular methods to accurately document heating‐induced changes, and the data indicate variation in the effects that heating has on individual pecan allergens. The appearance of full‐length Car i 2 and Car i 4 proteins was severely reduced at 20 and 24 min of heating, whereas Car i 1 appeared relatively unchanged based upon SDS‐PAGE and immunoblotting (Figure 1). These results are not surprising as longer heating times have demonstrated a reduction in Car i 2 and 4 (Mahesh Venkatachalam et al. 2006), whereas the 2S albumins, such as Car i 1, have been characterized as heat stable proteins (Murtagh et al. 2003; Bueno‐Díaz et al. 2021). Similarly, the Car i 1 peptide intensities observed by mass‐spectrometry were less affected by increased heating time relative to Car i 2 and Car i 4 (Figure 2). This is similar to what has been observed for Ana o 3, the orthologous 2S albumin, in heated cashew nuts (Mattison et al. 2016; Chen and Downs 2022). However, in cashew nuts, the Ana o 1 vicilin allergen is relatively more heat labile compared to the Ana o 2 legumin and Ana o 3 2S albumin (Mattison et al. 2016; Chen and Downs 2022).

The differences observed via SDS‐PAGE and immunoblotting of larger molecular mass proteins, such as Car i 2 and Car i 4 observed here and by others (Mahesh Venkatachalam et al. 2006), are potentially due to heating‐induced protein precipitation and insolubility, breakdown into smaller peptide fragments of less than 25 kDa, or both. Heating‐induced reduction in antibody binding with rabbit anti‐pecan sera or IgE from pecan allergic volunteers (Figure 2) was distinguished from the observation of numerous peptides by mass‐spectrometry, which indicated that much of the allergen content remained. The effects of heating are likely to be allergen specific, and the data presented here suggest that Car i 4 may be more prone to heating‐induced aggregation and/or precipitation and that Car i 2 may be more likely to fragment into smaller peptides. In support of this, there was relatively less change in the number of trypsin‐digested Car i 2 peptides observed by mass spectrometry throughout heating (Figure 4). In contrast, observation of Car i 4 peptides varied more after heating at 20 and 24 min suggesting that the protein was precipitated and unavailable for trypsin digestion and peptide detection. A similar occurrence has been noticed in heated soy samples where the basic soy legumin subunit has been observed to be more susceptible to heating‐induced precipitation (Utsumi, Damodaran, and Kinsella 1984). Further, soy vicilin and legumin allergens have been shown to associate and form large macro‐complexes (over a million kDa) after heating (Utsumi, Damodaran, and Kinsella 1984), which could prevent entry into SDS‐PAGE during electrophoresis and alter trypsin digestibility for mass‐spectrometry. To summarize the findings in Figures 3 and 4, the change in peptide abundance relative to peptide quantity demonstrates that no one allergen behaves identically to another following processing. In addition, the variation in heat lability observed between Car i 1, Car i 2, and Car i 4, makes the study of heating‐induced changes to protein content difficult. This highlights the importance of selecting reliable peptide markers to track the changes in protein composition and stability occurring in response to processing.

Peptide markers can be used as high confidence targets to track the presence of allergens and potentially reveal changes in heating‐induced allergen solubility, modification, and IgE binding capacity in pecan allergic individuals. A reliable peptide marker must meet certain criteria including: (1) specificity to the food/source, (2) resistance to processing, (3) production of MS peaks that are amenable to peak area calculations for easy quantification on different mass analyzers, and (4) observed at all time points with no missed cleavages of arginine or lysine residues. Although pecan allergen sequences share 97% homology with walnut (Elizur et al. 2020), we identified one Car i 1, six Car i 2, and six Car i 4 potential marker peptides after application of our criteria (Table 1). The peptides highlighted here remained relatively stable to heating, and the results of this study suggest that in general Car i 1 and Car i 2 derived peptides are likely more reliable markers than those derived from Car i 4. As shown in Table 1, select peptide markers were shown to coincide with strongly reacting IgE epitopes established by Sharma and others (Sharma et al. 2011a). Because this information can be useful in assessing changes in allergen potency after heating, an investigation of the observed Car i 1, Car i 2, and Car i 4 peptides relative to IgE epitopes was explored as these modifications may alter IgE binding.

Statistical analyses and protein modeling techniques have been used to approximate the location of IgE epitopes in proteins that commonly cause allergies. For example, in silico analysis was used to estimate the location and sequence of linear Car i 2 epitopes (Minkiewicz, Mattison, and Darewicz 2022). The results of this type of analysis along with experimentally assigned IgE epitopes were used to highlight the location of IgE epitopes in treated samples. Comparison of heating‐induced peptide modifications with published linear IgE epitopes revealed some overlap (Table 2). The Car i 1 peptide, “QQQQEEGIRGEEMEEMVQCASDLPKECGISSR”, contained two missed cleavages (R111 and K127), was modified at K127, and harbored two linear peptides (“QEEGIRGEEMEE” and GIRGEEMEEMVQ) that reacted strongly with pecan allergic IgE (Sharma et al. 2011a). From Car i 2, two sequences, “LEMVKLLQPVNNPGQFR” and “QLQEMDVLVNYAEIKR”, each showed one missed cleavage, contained fragments that overlapped IgE binding sites (“PGQFR” and “QEMDVLVNYAEIKR” in Jug r 2), and sustained modifications other than hydroxymethylOP and delta:H(2)C(3)O (1). Similarly, the Car i 4 peptide “EGQVLTVPQNFAVIKK” contains one missed cleavage and includes a potentially immunoreactive sequence “PQNFAV” that shares homology with the Jug r 4 epitope “PQNFGV”, which only differs by a single amino acid. However, since much of the protein remains for Car i 1 and Car i 2, and Car i 4 (to a lesser extent) following heat treatment, these data support previous findings demonstrating that heating‐induced modification may alter immunoreactivity via the formation of heat stable peptides of greater length due to missed cleavages resulting from heating‐induced modification of trypsin cleavage sites (Verhoeckx et al. 2015; Vissers, Blanc, et al. 2011, Vissers, Iwan, et al. 2011; Mattison et al. 2015; Astuti et al. 2022). Although no immunological consequence has been demonstrated, a heating‐induced modification of the arginine 111 (R111) residue in the cashew 2S albumin Ana o 3 has been described (Mattison, Grimm, et al. 2017).

Heating‐induced arginine and lysine modifications on Car i 1, Car i 2, and Car i 4 were identified in this study (Table 2). The most abundant arginine or lysine modifications observed in descending order include delta:H(2)C(3)O (1), hex, hydroxymethylOP, carboxyethyl, carboxyethyl, carbamyl, G‐H1, and propionamide modifications (Figure 5). Methionine oxidation is observed in both unheated and heated samples. It is important to point out that methionine oxidation accounts for up to 38% of all modifications occurring in heated samples, suggesting a potentially useful molecular marker for pecan aging during storage.

**FIGURE 6:**
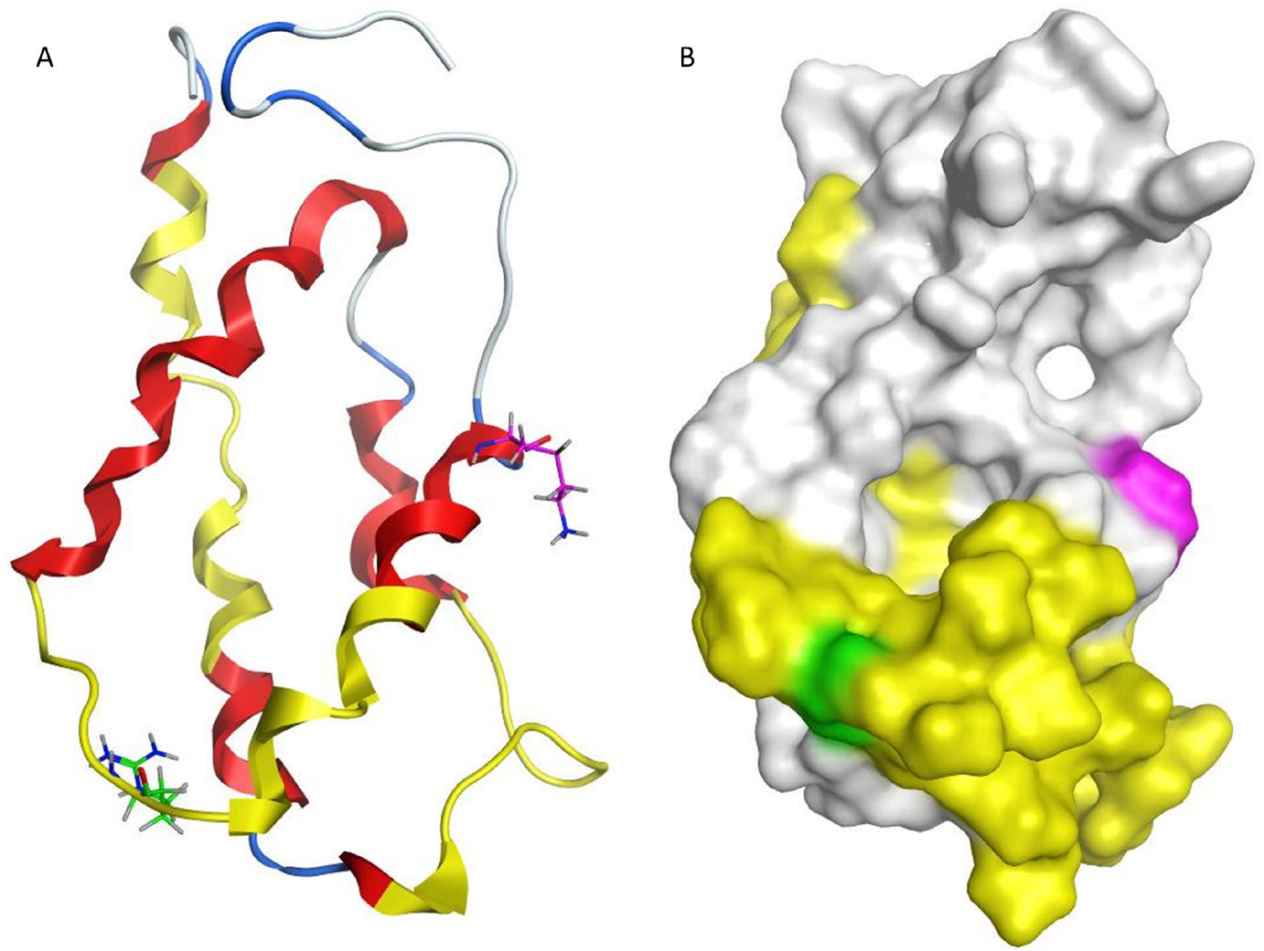
Highlighting heating‐induced modifications and linear Car i 1 IgE epitopes. Ribbon (A) and surface (B) models of Car i 1 depicting heating‐induced modification sites at R111 (green) and K127 (magenta), with strongly reacting IgE epitopes (yellow) (Sharma et al. 2011a), alpha helices (red), loops (blue), and unstructured regions (gray).

Based on the cumulative data presented here, Car i 1 remained almost entirely soluble during the heating treatments, and numerous Car i 1 modifications, including hydroxymethylOP, delta:H(2)C(3)O (1) (R), and carboxyethyl, were specifically observed only on the heated samples. These observations could provide support for altered Car i 1 allergen potency following heating. Modeling of the Car i 1 allergen highlights the overlap between the heating‐induced R111 modification and a linear Car i 1 IgE epitope (Sharma et al. 2011a) (Figure 6).

While the R111 residue in Car i 1 is conserved in Ber e 1, Jug r 1, and Jug n 1, it aligns with K106 in Ana o 3, but not with the modified R111 residue that has been observed in Ana o 3 (Mattison et al. 2017a). Conversely, the K127 Car i 1 residue is not well conserved with other 2S albumins and does not overlap with any reported IgE epitopes. Heating‐induced protein modifications on surface residues such as arginine and lysine may also result in altered allergen immune reactivity and recognition by IgE antibodies. For example, heating‐induced modifications on arginine or lysine residues can prevent digestion by trypsin resulting in a relatively more stable allergen (Mattison et al. 2015; Mattison, Rai, et al. 2017b; Mahesh Venkatachalam et al. 2006).

It remains unclear how chemical modification of individual amino acids may impact food allergen potency. For example, while heating‐induced modifications on lysine or arginine can destroy trypsin cleavage sites and thereby render a peptide or protein more stable (Mattison et al. 2015), we are not aware of demonstrations indicating that these modifications can directly alter (increase or decrease) the affinity of IgE antibody binding to specific food allergens. While other allergens are degraded by heating, it is clear that some food allergens such as the 2S albumins are heat stable, and this may in part explain why they have greater potency in assays measuring effector activity in cells (Hazebrouck et al. 2022; Porterfield et al. 2009; Sanchiz et al. 2017; Zhuang and Dreskin 2012). The continued identification of marker peptides, immune reactive protein residues, and heating‐induced modifications within food allergens is integral to understanding the relationships between processing and allergen content or potency observed between heated and unheated pecans and other foods.

## Author Contributions

**C. Nacaya Brown:** investigation (equal), writing – original draft (equal), writing – review and editing (equal). **Rebecca A. Dupre:** data curation (equal), formal analysis (equal), methodology (equal), writing – original draft (equal), writing – review and editing (equal). **Christopher C. Ebmeier:** data curation (equal), methodology (equal), writing – review and editing (equal). **Shaina Patil:** data curation (equal), writing – review and editing (equal). **Brennan Smith:** funding acquisition (equal), resources (equal), writing – review and editing (equal). **Christopher P. Mattison:** conceptualization (equal), formal analysis (equal), investigation (equal), project administration (equal), supervision (equal), visualization (equal), writing – original draft (equal), writing – review and editing (equal).

## Ethics Statement

This study does not involve any human or animal testing.

## Consent

Plasma Lab International (Everett, WA, USA), is a United States Food and Drug Administration (FDA) licensed facility (# 1050). Informed consent was obtained from each volunteer donor, and all participants (or their proxies/legal guardians) provided written informed consent for the publication of their anonymized case details. Plasma samples were collected according to the US‐FDA Code of Federal Regulations Title 21 by apheresis using an Aurora collection instrument with the inclusion of sodium citrate as an anticoagulant.

## Conflicts of Interest

The authors declare no conflicts of interest.

## Supporting information


Table S1.

Table S2.

## Data Availability

Data available in article Supporting Information and available on request from the authors.
