# Supplementary material for: Heating Differentiates Pecan Allergen Stability: Car i 4 Is More Heat Labile Than Car i 1 and Car i 2
Source: Food Sci Nutr. 2025 Feb 16;13(2):e4747. doi: 10.1002/fsn3.4747 (PMC11830569; doi:10.1002/fsn3.4747)
Supplement: Supplementary file 1 — Table S1. [file FSN3-13-e4747-s001.docx]

Supporting Information

Heating differentiates pecan allergen stability: Car i 4 is more heat labile than Car i 1 and Car i 2

C. Nacaya Brown ^1,2^, Rebecca A. Dupre ^1,2^, Christopher C Ebmeier ^3^, Shaina Patil ^1^, Brennan Smith ^1^, Christopher P Mattison ^1*^

^1^ Food Processing Sensory Quality, USDA Agricultural Research Service, New Orleans, LA, 70124, USA

^2^ Oak Ridge Institute for Science and Education, U.S. Department of Energy, Oak Ridge, TN 37831, USA

^3^ Department of Biochemistry, University of Colorado Boulder, Boulder, CO, USA

* Correspondence: chris.mattison@usda.gov; Tel.: +1 (504) 286-4392

Supplemental Table #1. Variable modifications included in Mascot searches of LCMS data. A custom pecan allergen library was used to search for the proteins of interest. Cysteine carbamidomethylation (delta m/z 57.021464) was set as a fixed modification and methionine oxidation (delta m/z 15.994915) was set as a variable modification in all searches. Preliminary error tolerant searches were done to identify potential lysine and/or arginine modifications. Searches were performed in three iterations to include the variable modifications listed

| Search group (iteration) | Unimod name, PSI-MS or Interim | Formula | Delta m/z, monoisotopic | Modification site |
| --- | --- | --- | --- | --- |
| 1 | Hex | C_6_H_10_O_5_ | 162.052824 | KR |
| 1 | HydroxymethylOP | C_6_H_4_O_2_ | 108.021129 | K |
| 1 | Propionamide | C_3_H_5_NO | 71.037114 | K |
| 2 | G-H1 | C_2_O | 39.994915 | R |
| 2 | Carboxymethyl | C_2_H_2_O_2_ | 58.005479 | K |
| 2 | Carboxyethyl | C_3_H_4_O_2_ | 72.021129 | K |
| 3 | Carbamyl | CHNO | 43.005814 | KR |
| 3 | Delta:H(2)C(3)O(1) | C_3_H_2_O | 54.010565 | KR |
